# Short-Term Effects of Suspension Training on Strength and Power Performances

**DOI:** 10.3390/jfmk3040051

**Published:** 2018-10-23

**Authors:** Giuseppe Francesco Giancotti, Andrea Fusco, Alice Iannaccone, Cristina Cortis

**Affiliations:** 1 Department of Human Sciences, Society and Health, University of Cassino and Lazio Meridionale, 03043 Cassino, Italy; 2 Department of Sports Science and Kinesiology, University of Salzburg, 5400 Salzburg, Austria

**Keywords:** instability, jump, maximum voluntary contraction, handgrip

## Abstract

Suspension Training (ST) workouts include a variety of movements requiring the individual to maintain balance while performing various resistance exercises in an interval fashion. Although ST is thought to elicit higher muscle activations than traditional exercises, only limited information is available on its acute effects on strength and power performances, especially in relation to gender. Therefore, the purpose of this study was to evaluate the strength and power acute responses after ST, also in relation to gender. Eighty-eight (46 males, 42 females) participants were administered countermovement jumps (CMJ), squat jumps (SJ), lower limb Maximum Voluntary Contraction (MVC) at 90° angle knee extension, and grip strength (handgrip) before (PRE) and after (POST) a 50 min ST session involving upper, lower body and core exercises. ANOVA for repeated measures was used to evaluate the differences (*p* < 0.05) in relation to gender and experimental session. After ST session, significantly higher values emerged in males, whereas no significant changes were found in females. Findings indicate that ST as a form of exercise is useful to maintain and improve acute strength and power performances, especially in male participants. Future studies should be carried out to explore the gender-related differences in response to acute bout of ST exercises.

## 1. Introduction

Body weight resistance training is an inexpensive way to exercise effectively [1], with suspension training (ST) promoting multi-planar and multi-joint bodyweight exercises using two independently moving handles suspended by two straps with a fixed anchor position above the exerciser [2,3,4,5]. The load imposed by ST exercises is determined by three fundamental principles: vector-resistance (the inclination of the exerciser’s body relative to the ground determines the intensity of the exercise), stability (the size and positioning of the base of support relative to the centre of gravity determines the stability of an exercise), and pendulum (the positioning of the centre of gravity relative to the anchor point determines the intensity of the exercise) [4,6,7]. Based on these principles, it is possible to regulate the resistance by changing the angle, the base of support and the distance from the anchor point, making ST as a valuable form of resistance training which is easily portable and inexpensive. 

Considering the popularity of ST, several studies were performed in the last 5 years to investigate the cardiovascular [8,9], neuromuscular [10,11,12,13,14] and biomechanical [3,7,15] characteristics of this activity. The unstable nature of the base of support characterizing ST has been reported to stress the neuromuscular system with greater muscular demands than traditional resistance exercises performed on stable surfaces [13,14,16,17,18] with subsequent increases in strength, attributable to changes in muscle cross-sectional area and neuromuscular coordination [13,19]. Therefore, acute [8,20] and chronic [21,22,23,24] improvements in strength, endurance, flexibility, functional and core stability have been reported in relation to ST, with the majority of studies focusing solely on either male or female participants and not addressing the gender-related differences. In particular, in previous studies assessing the acute effects of ST, short-term increases in oxygen consumption and reductions in isometric force production have been shown in healthy college-aged males after a ST session involving specific exercise only (i.e., 2 and 4 suspended limbs push-ups performed for 6 sets of 10 repetitions) [25]. Moreover, studying the acute effects of ST on muscle activation, resistance load, and fatigue, great isometric force decrements have been reported in males performing biceps curls to fatigue [26]. Finally, investigating the effects of a ST warm-up on throwing velocity and accuracy, sport-specific acute improvements in strength, power and velocity of execution emerged [20]. Conversely, only a few studies have investigated the effects of ST sessions in females, showing chronic improvements of strength in recreationally active young women [21], and throwing performance parameters (i.e., throwing velocity, the mean external rotation power or the mean peak power in shoulder flexion) in National Collegiate Athletic Association Division I softball players [22].

To the best of our knowledge, only one study [27] included a whole ST session to investigate the acute anabolic hormonal responses to an acute bout of ST, showing that 30 s work intervals with 60 s rest periods session elicited typical total testosterone and novel testosterone to cortisol ratio responses in physically active males.

Therefore, as only limited information is available on ST short-term effects on performances, especially in female participants, the aim of the present study was not only to evaluate strength and power acute responses after group ST, but also to examine the gender differences in relation to ST. 

## 2. Methods

### 2.1. Experimental Design 

This study was designed to investigate the changes in strength and power performances in relation to a group ST session. After three experimental sessions designed to familiarize the participants with the entire protocol and to avoid any learning effect, participants took part in a 50 min group ST session, consisting of a warm-up phase, exercises for core, upper and lower limb muscles and a cool-down phase as reported in Table 1. The exercises included in the session were as follows [28]:

- Suspended plank: feet (toes down) in foot cradles under the anchor point and forearms on the floor, keeping the body aligned for the planned time, without letting the hips or the back sag;
- Sprinter’s start knee-up: behind the ST device, grasping the handles with the straps underarms, bringing the rear knee up and forward, alternating the legs;
- Chest press: behind the ST device, grasping the handles with extended arms, lowering the chest in push-up motion and returning to the start position;
- Pike: starting from the suspended plank position, flexing at the hips slowly and under control until shoulders, hips, and legs are on a 90° angle and returning to the start position;
- Single leg squat: standing facing the ST device, grasping the handles with elbows bent at sides and extending the right leg forward, lowering the pelvis toward the floor and returning to the start position, alternating the legs;
- T deltoid fly: standing facing to ST device, grasping the handles with extended arms, extending both arms to make a T shape pulling the body upright and keeping tension in the ST straps, then return to the starting position;
- Oblique crunch: starting from the suspended plank position, lifting the pelvis and bringing the knees to the right elbow, returning to the starting position and bringing the knees to the left elbow;
- Squat jump: standing facing the ST device, grasping the handles with the elbows bent at each side, lowering the pelvis toward the floor and return to the starting position, performing a jump at top of the movement;
- Back row: stand facing the ST device, grasping the handles with extended arms, pulling the chest forward with a 45° angle of the body with respect to the ground and then returning to the start position.

In line with a similar research studying the acute effects on performance parameters [29,30,31,32,33], before (PRE) and after (POST) the ST session, power performances were evaluated by the means of squat (SJ) and countermovement (CMJ) jumps, whereas strength performances were evaluated by the means of maximum voluntary contraction (MVC) and maximum isometric grip force (handgrip). Previous findings suggested that distinct neural control mechanisms are employed for dominant and non-dominant arm movements [34] and that differences exist between dominant and non-dominant legs [35]. Likewise, in the present study, MVC and handgrip parameters were collected and analyzed also in relation to limb dominance. Data collected during the second and the third familiarization session were used to assess the reliability of the measurements. Good to excellent test-retest intraclass correlation coefficients (ICCs) were reported for SJ (0.93), CMJ (0.95), MVC (range: 0.64–0.76), and handgrip (range: 0.85–0.95).

### 2.2. Participants

Eighty-eight (46 males: age = 26.6 ± 4.4 years; body mass: 72.5 ± 9.1 kg; height: 173.2 ± 5.9 cm; 42 females: age = 24.8 ± 2.6 years; body mass: 57.5 ± 7.8 kg; height: 159.9 ± 6.3 cm) physically active (engaging in at least 3 days·week^−1^ of moderate-to-intense physical activity) college students volunteered to participate in the study. Participants were included if they reported ST experience (at least 1 session weekly in the past year) and they didn’t report any pre-existing condition such as recent physical injuries, musculoskeletal disorders, cardiovascular, respiratory and/or metabolic diseases. Written informed consent was obtained from all participants in accordance with the declaration of Helsinki for human research of 1964 (last modified in 2000) and the study was approved by the local Institutional Review Board (approval number: 17845.2018.09.12; date: 20 September 2018). 

Prior to the beginning of ST session, body weight and height were measured using a scale with integrated stadiometer with a precision of 0.1 kg and 0.1 cm (Seca, model 709, Vogel & Halke, Hamburg, Germany). Leg dominant was determined by asking each participant the favorite foot to kick a ball, while the hand dominant by asking the hand favorite to throw a ball [36].

### 2.3. Performance Evaluations

Jump tests were evaluated by means of a portable force plate (Kistler Quattro Jump 9290AD, Kistler, Winterthur, Switzerland; range from 0 N to 10,000 N; linearity < ±0.5% FSO) according to the previous protocols [37,38,39]. 

For SJ, starting from the upright standing position with their hands on their hips, participants were instructed to flex their knees and hold a predetermined knee position (approximately 90°). The experimenter then counted out loud for 3 s and on the count of 3, the participants were instructed to jump as high as possible without performing any countermovement before the execution of the jump. Each invalid trial (i.e., if some movement was perceived that might have increased the flexion of knees when starting the jump), was repeated. For CMJ, starting from the upright standing position with their hands on their hips (i.e., without arm swing), participants were instructed to flex their knees (approximately 90°) as quickly as possible and jump as high as possible in the ensuing concentric phase. Each invalid trial (i.e., if the knees were not bent to maximum intensity and quickly without a break or the course of movement was inverted through the extension of the knees), was repeated.

For both jumps, participants were required at takeoff to leave the floor with their knees and ankles extended and land in a similarly extended position. They were also instructed to land in the same point of takeoff and to rebound with straight legs when landing, to avoid knee bending and alteration of measurements. Moreover, to avoid any discomfort when landing, participants were encouraged to repeatedly bounce on the tip of the toes with their knees fully extended.

MVC at approximately 90° angle isometric knee extension was measured with participants seated in a customized chair with arms crossed on the chest and they were asked to exert a force by extending the knee. The test consisted of a force-rising phase, a sustained phase and a relaxation phase. When the produced force reached a plateau in the sustained phase, the participants were encouraged by the supervisors to obtain a further increase in knee extension force. MVC was measured 3 times in dominant and non-dominant limbs with a 1 min rest in between. Data were collected by a piezoelectric force transducer (Kistler 9321B, Kistler, Winterthur, Switzerland; range from −10,000 N to 10,000 N; sensitivity ≈ −4 pC/N; linearity ≤ ±0.5% FSO) connected with a data acquisition system (Tektronix TBS 1202B, Tektronix, Beaverton, OR, USA) by means of a charge amplifier (Kistler 5001, Kistler, Winterthur, Switzerland). 

Handgrip was measured in dominant and non-dominant limbs with an adjustable hydraulic hand dynamometer (Jamar Hand Dynamometer 5030J1, Sammons Preston, Bolingbrook, IL, USA; ranging from 0 kg to 90 kg, data subsequently expressed in Newton). From a standing position, shoulders adducted and neutrally rotated, elbow flexed at 90°, forearm in neutral position and wrist between 0° and 30° of flexion and between 0° and 15° of ulnar deviation, participants were instructed to squeeze the dynamometer with the maximum isometric effort maintained for 3 s three times with 1 min rest in between.

For each strength and power performance test, the best out of three measurements was used for the subsequent statistical analysis.

### 2.4. Statistical Analysis

Data normal distribution was verified by the Shapiro-Wilk test and the means and standard deviations were calculated for each of the analyzed variables. The criterion for significance was set at an alpha level of *p* < 0.05. Statistical analyses were conducted using the statistical package Stata statistical software version 14.1 (Stata-Corp, College Station, TX, USA). A two (gender: male vs. female) by two (limb dominance: dominant limb vs. non-dominant limb) by two (experimental session: PRE vs. POST) repeated-measures analysis of variance (ANOVA) was used to test for differences in MVC and handgrip data, whereas a two (gender: male vs. female) by two (experimental session: PRE vs. POST) repeated-measures ANOVA with post hoc comparisons was used to test for differences in CMJ and SJ data. If significant interactions and main effects emerged, post hoc comparisons were performed by the means of Fisher’s Least Significant Difference with Bonferroni alpha level correction to eliminate an inflated Type 1 error for multiple comparisons. Furthermore, to provide meaningful analysis for comparisons from small groups, the Cohen’s effect sizes (ES) were calculated. An ES of 0.2 or less was considered trivial, from 0.3 to 0.6 small, less than 1.2 moderate, and greater than 1.2 large [40].

## 3. Results

PRE and POST values for CMJ, SJ, MVC and handgrip performances in relation to gender have been reported in Table 2.

For CMJ performances, main effects were found for gender (*p* < 0.0001) with better performances in males, experimental session (*p* = 0.004) with increased performances in the POST condition, and the interaction gender by experimental session (*p* = 0.03), with post hoc analysis showing higher POST values in males only (*p* = 0.0007; ES = 0.3). Regarding SJ performances, main effects were found for gender (*p* < 0.0001) with better performances in males, experimental session (*p* = 0.01) with increased performances in the POST condition, and the interaction gender by experimental session (*p* = 0.03), with post hoc analysis showing higher POST values in males only (*p* = 0.009; ES = 0.3).

For MVC, main effects were found for gender (*p* < 0.0001) with better performances in males, limb dominance (*p* = 0.0001) with higher performances in the dominant limb, and experimental session (*p* = 0.0002) with increased performances in the POST condition. Post hoc analysis showed higher POST values in dominant (*p* = 0.002; ES = 0.4) and non-dominant (*p* = 0.01; ES = 0.3) limbs in males only. Regarding the handgrip data, main effects were found for gender (*p* < 0.0001) with better performances in males, limb dominance (*p* < 0.0001) with higher performances in the dominant limb, experimental session (*p* = 0.04) with increased performances in the POST condition, and the interaction gender by limb dominance by experimental session (*p* = 0.03), although after the Bonferroni correction, no significant differences emerged.

## 4. Discussion

The aim of the present study was to evaluate the short-term effects of ST on strength and power performances, also in relation to gender. The main findings of the present study were that ST elicited acute improvements in strength and power performances in males only.

In line with the literature [41,42], regardless of the experimental condition, males showed better performances with respect to females. After the ST session, gender-related acute improvements in strength and power were observed. In particular, males reported higher values in SJ, CMJ, and MVC (regardless of limb dominance), whereas no changes emerged in females. 

Contrasting results have been reported investigating the short-term effects of ST exercises, with a study showing sport-specific acute improvements in strength, power and velocity of execution [20], and others reporting reductions in isometric force production [25,26]. Although after an acute bout of exercise, a deterioration in performance could be expected due to fatigue [43], exercise-related arousing effects have been reported in complex motor behaviors regardless of age and expertise [29,30,31,32,33,44]. Therefore, the acute improvements in jump performances found in males after the ST session (CMJ: +4.6%; SJ: +4.3%), could be attributed to the increases in psychomotor speed and central executive control functions exerting a preservative effect on the efficiency of those executive attentional processes involved in the control of complex motor behavior [45]. Jump performances represent a complex coordinative task resulting from the interaction between the force of contraction and speed of movement (i.e., power), thus, it is likely that CMJ and SJ performances could be more affected by acute bouts of exercise than static strength [46]. In fact, acute reductions (or no changes) in isometric force production have been reported after ST exercises and sports competitions [25,26,29,30,32], in which the authors attributed to fatigue due to the specific involvement of the limbs during the activity. As ST session used in the present research included both upper and lower limbs exercises, similar findings could have been expected, with decrements or no changes in upper and lower limbs strength production. Conversely, increases in strength performances (MVC) were found, although gender differences emerged. In particular, males showed significant increases in MVC (dominant limb: +7.7%; non-dominant limb: +6.1%), whereas no effects emerged in females. During ST sessions, the evaluation of the load distribution during each exercise could be affected by several biomechanical aspects, such as the body inclination, the length of ST device, the feet position, and the combination of these factors [3,7,15]. Therefore, it is possible that participants performed each exercise in the most comfortable position, thus leading to exercise-related arousal rather than fatigue in strength parameters. Moreover, ST could be classified as moderate-to-vigorous intensity exercise [6,8] and the nature of the lesson used in this study (designed to alternate exercises for core, lower and upper body on the unstable surface) stressed the neuromuscular system providing an exercise intensity and duration exerting arousing effects on performances in lower limbs. As while performing most of the ST exercises included in the lesson, the participants are required to isometrically hold the two moving handles even when performing dynamic exercises, it could be speculated that the isometric activity did not exert any beneficial arousing effect on grip strength.

Females showed no significant changes in performances. Similar findings were found for power athletes [47], in which studying the gender-related acute effects of resistance exercise on strength and power performances among other factors, no gender differences emerged investigating the acute effects of performing back squats on subsequent performance [48]. As the existing literature mainly focused on males or reported non-conclusive findings, it could only be speculated that female participants did not benefit from the exercise-related arousing effects observed in males. For MVC, males showed improvements regardless of limb dominance, whereas no significant change emerged in females. Although the literature reported gender-related differences in acute response to exercise due to muscular fatigue [49,50], it seems that short-term effects after ST are somehow limb-specific. In conclusion, the current results indicate that ST is a form of exercise exerting short-term increases in strength and power performances in male participants. Considering that no reductions emerged in both strength and power performances regardless of gender, ST could also be used in different warm-up exercises in sports and activities where these characteristics play a key role. However, future studies are encouraged to further explore the gender-related differences in response to an acute bout of exercise, as the results are still not exhaustive. 

## Figures and Tables

**Table 1:** Schematic Representation of the 50 min Suspension Training Group Session.

Sets	Exercise	Duration
	WARM-UP	8′
3 x	Suspended plank	45″
	Sprinter’s start knee-up	45″
	Chest press	45″
	RECOVERY	3′
3 x	Pike	45″
	Single leg squat	45″
	T deltoid fly	45″
	RECOVERY	3′
3 x	Oblique crunch	45″
	Squat jump	45″
	Back row	45″
	RECOVERY	3′
	COOL-DOWN	8′

**Table 2:** Strength and Power Performances Before and After the 50 min Suspension Training Group Session in Male and Female Participants.

		Males		Females	
Variable		*Pre*	*Post*	*Pre*	*Post*
Jumps (cm)	CMJ	32.5 ± 4.4	34.0 ± 4.8 *	19.4 ± 3.5	19.4 ± 3.5
	SJ	28.2 ± 3.9	29.4 ± 4.1 *	17.3 ± 3.7	17.3 ± 3.5
MVC (N)	Dominant limb	280.9 ± 59.4	302.5 ± 57.4 *	238.8 ± 46.5	255.0 ± 41.7 *
	Non-dominant limb	277.0 ± 52.8	294.0 ± 56.3 *	225.6 ± 49.0 #	238.5 ± 49.5
Handgrip (N)	Dominant limb	423.8 ± 79.7	440.4 ± 69.5 *	256.0 ± 44.9	257.9 ± 43.2
	Non-dominant limb	398.9 ± 54.1 #	405.3 ± 52.2	233.3 ± 46.5 #	239.2 ± 46.8

CMJ: countermovement jump; SJ: squat jump; MVC: maximum voluntary contraction. * denotes significant (*p* < 0.05) differences from pre values; # denotes significant (*p* < 0.05) differences form dominant limb values.
